# Extinction: A synthesis of disciplines for theoretical and practical advances

**DOI:** 10.1017/ext.2023.2

**Published:** 2023-01-12

**Authors:** Barry W. Brook, John Alroy

**Affiliations:** 1 School of Natural Sciences, University of Tasmania, Hobart, TAS, Australia; 2 Department of Biological Sciences, Macquarie University, Macquarie Park, NSW, Australia

**Keywords:** Extinction, Biotic interactions, Biodiversity conservation, Population processes, Community ecology

## Abstract

*Extinction* is a new open-access journal focused on the patterns and processes underlying the loss of biodiversity. It aims to inform conservation efforts, with a broad spatial and temporal scope. Extinction biology – the scientific study of species loss – has a long history and has recently become a more interdisciplinary and integrated field. This journal offers a unique, synthetic forum in which to present cutting-edge research and discuss its implications. This includes ecological, molecular, paleontological, and social perspectives, based on empirical data, theory, and modelling, to understand extinction processes. By tackling the big challenges, the research published in *Extinction* will be valuable for researchers and practitioners concerned with extinction and its role in shaping the history and future of life on Earth.

Cambridge Prisms journals capture the fast-paced evolution of scientific research in the 21st century. The new title *Extinction*, for which we are joint Editors-in-Chief, documents species extinction, diversity loss, population processes, mass extinction, and human factors. Its scope embraces empirical and theoretical studies at all spatial and temporal scales, ranging from deep time to the present. The goal is not only to link patterns to mechanisms, but to better inform the protection of global biodiversity.

Extinction biology is a newly defined scientific discipline, but its history can be traced back to the early 19th century, when scientists first recognised that species could disappear forever. At that time, the French naturalist Cuvier (1818) used fossil evidence to demonstrate that many species are no longer present on Earth. By the mid-19th century, Darwin (1859) provided a scientific explanation for the phenomenon: species that are unable to adapt to changing environmental conditions are more likely to go extinct. As research on natural history expanded over the coming decades, a divide came to separate those who studied the facts of extinction as documented in the fossil record and those who investigated contemporary threats to organisms. Palaeontologists focused on discovering extinct lineages, while conservationists concentrated at first on raising the alarm over hunting and related threats. The rapprochement between palaeontologists and evolutionists during the Modern Synthesis of the mid-20th century (Huxley, 1942) did little to bridge the divide. In recent years, however, there has been a growing recognition that both the causes and consequences of extinction, past and present, must be understood to effectively conserve biodiversity. As a result, the study of extinction has become more integrated and interdisciplinary.

Efforts to bring the field of extinction biology together and promote collaborative research have included symposia and themed journal issues. One example is the International Union for Conservation of Nature’s World Conservation Congress,^1^ which has gathered researchers from ecology, conservation biology, and palaeontology to discuss the latest research on extinction and its impacts on biodiversity. Past conferences and special issues (e.g., Brook and Alroy, 2017) have highlighted the role of habitat destruction in species extinction, the impacts of climate change on extinction, and the use of evolutionary biology to inform conservation efforts. These conferences have also featured discussions on the ethical and social implications of extinction and the importance of promoting conservation efforts. However, these efforts have been sporadic, and there has never been an ongoing forum for extinction biology *per* *se*.

Analytical methods are a particularly important locus for synergy in extinction biology. One general example is the fusion of molecular and paleontological data. For example, fossil evidence is crucial not only to calibrating molecular phylogenies but to inferring net diversification rates from molecular data. Meanwhile, information on the genetic makeup of particular species such as woolly mammoths, combined with paleontological data, can paint a more complete picture of the extinction processes (Fordham et al., 2021). Another example is the use of modelling and empirical data to study ongoing extinctions and to develop a more nuanced view of causes and consequences.

Recent work has illustrated the importance of indirect effects, such as the way habitat destruction can lead to changes in such interactions as competition for resources and predator–prey dynamics (Rybicki et al., 2020). At large scales, documenting mass extinctions in the deep fossil record has helped to contextualise the unfolding biodiversity crisis, driven this time by human factors. By looking at past events, which have involved such processes as global climate perturbations and human hunting, the precise mechanisms which lead to contemporary biodiversity loss and subsequent impacts on human welfare can be better resolved.

*Cambridge Prisms: Extinction* is a milestone in the progress of disciplinary integration. As a cross-disciplinary journal, it brings together research from a range of scientific fields, including ecology, evolution, conservation biology, palaeontology, as well as the humanities, to provide a comprehensive view. Its goal is not only to disseminate research coming from these subdisciplines, but to integrate multiple perspectives and approaches on a case-by-case basis. By publishing high-quality, open-access research on all facets of the topic, *Extinction* can help educate the public and promote awareness of the need, and the evidence-based options available, to protect global biodiversity.

However, there are still many challenges ahead for the journal and for the broader field of extinction biology. Scientific issues are related to both data and methods. For instance, because rare species are more likely to go extinct, relevant data are often scarce. Indeed, this problem motivates the large literature on statistical inference of extinction based on records of individual species that goes back to Strauss and Sadler (1989). Meanwhile, there has long been much debate over how to quantify ensemble extinction rates in the fossil record (Foote, 1994). Estimating the number of species that are going extinct right now is problematic because key approaches, such as interpolating species-area curves or applying species distribution models, may lack realism. In general, the processes involved in extinction are often complex, so they must be studied using innovative, integrative methods.

The variable quantity and quality of relevant data is another major challenge (Peters, 1991). In particular, there is a need for more comprehensive and accurate data on the distribution and abundance of species and communities, both extinct and extant. Point-location data based on field surveys of communities and observations of individual species provide essential information. Yet these records are sparse for most species and their quality depends on the methods used to collect and analyse the data. Data on the composition and structure of species communities, including species richness, relative abundances, and spatial turnover, are similarly variable in coverage and quality. In some regions, there is extensive long-term monitoring, while other regions are an information vacuum. This challenge must continue to be tackled.

Improving data relevant to extinction will involve supporting museum collections, citizen science, and scientific exploration and monitoring. Specimens in natural history museums provide valuable information on the distribution of individual species. Public participation in scientific research also provides valuable data of this kind. Ecological and palaeontological surveying adds another dimension, by documenting community-wide patterns. Data on the tropics are of particular concern for extinction biology because this part of the world hosts such a vast amount of biodiversity and is particularly vulnerable to habitat destruction and other extinction drivers (Sodhi and Brook, 2006). Thus, research in the tropics demands greater support.

Going beyond data and methods, a major challenge for extinction biology is the need to communicate both the science and its practical value to policy makers and the public. Despite the importance of extinction as a global issue, many people are not well-informed about the scale of the current extinction crisis and the attendant, urgent need for conservation efforts. Presenting effective solutions is the ultimate goal for the subdiscipline, but communicating these solutions is easier said than done (Bickford et al., 2012).

*Cambridge Prisms: Extinction* represents the culmination of efforts to promote synergy between relevant disciplines. The diverse array of review articles profiled in its early issues will set the stage for future research, providing overviews of the latest advances that highlight the potential for cross-fertilisation and its value. They also provide a framework for future research by identifying gaps in current knowledge. By promoting interdisciplinarity and publishing cutting-edge research, the journal will both advance our understanding of extinction and support conservation efforts. And by tackling the big challenges in extinction biology, it will illuminate the role of extinction in shaping the history and future of life on Earth.

## Footnotes

1
https://www.iucn.org/our-union/iucn-world-conservation-congress.
